# Entropy as a marker of physiological transition during pediatric cardiopulmonary exercise testing

**DOI:** 10.3389/fphys.2025.1698399

**Published:** 2025-12-01

**Authors:** Kaleigh O’Hara, Donald E. Brown, Dan M. Cooper, Annamarie Stehli, Shlomit Radom Aizik, Natalie Kupperman

**Affiliations:** 1 School of Data Science, University of Virginia, Charlottesville, VA, United States; 2 Institute for Clinical and Translational Science, University of California, Irvine, CA, United States; 3 Department of Pediatrics, Pediatric Exercise and Genomics Research Center, University of California, Irvine, CA, United States

**Keywords:** entropy, pediatrics, cardiopulmonary exercise testing, breath-by-breath, bayesian statistics

## Abstract

This research analyzed the sample entropy (SampEn) of breath-by-breath cardiopulmonary exercise testing (CPET) data from 170 healthy pediatric participants (85 males) 8 to 18-years-old, using a Bayesian statistics approach. SampEn measures the complexity of time series data, providing quantitative insight into the predictability of breathing patterns in pediatric participants. To address non-stationarity, signals were differenced prior to SampEn calculation. In addition to sex and age group comparisons, we examined SampEn before and after the midpoint of each participant’s CPET to assess how SampEn changes as exercise intensity increases. We corroborated previous findings that SampEn decreases in the later half of CPET for healthy pediatric participants for oxygen uptake $\left(\dot{V}O_{2}\right)$ , carbon dioxide output $\left(\dot{V}CO_{2}\right)$ , ventilation $\left(\dot{V}E\right)$ , and heart rate (HR) . Females tended to have higher SampEn than their male counterparts, with a statistically significant difference between the sexes in older participants for $\dot{V}O_{2}$ , $\dot{V}CO_{2}$ , $\dot{V}E$ , HR , and respiratory rate (RR) . Age-related findings included: significantly higher SampEn in younger males compared to older males for $\dot{V}O_{2}$ and $\dot{V}E$ and older female participants had a higher SampEn in older females compared to younger females for HR. These findings support SampEn as a sensitive, non-invasive marker of physiological transition during pediatric CPET, with potential applications in exercise physiology research and clinical assessment.

## 1 Introduction

Cardiopulmonary exercise testing (CPET) is a non-invasive clinical tool with the ability to provide wide-ranging insights into human health for clinical applications (Myers et al., 2014; West et al., 2016; Smith et al., 2022; Cooper et al., 2023). During a progressive CPET, the intensity of exercise increases incrementally, typically on a treadmill or cycle ergometer, while physiological metrics such as heart rate (HR) , respiratory rate (RR) , tidal volume $\left(V_{T}\right)$ , oxygen uptake $\left(\dot{V}O_{2}\right)$ , carbon dioxide output $\left(\dot{V}CO_{2}\right)$ , and ventilation $\left(\dot{V}E\right)$ are continuously measured breath-by-breath. This amounts to substantial data collection. The duration of a typical pediatric CPET is about 8–12 min, with an average respiratory rate of 25 breaths per minute generating approximately 250 breaths. At each of these breaths, individual values for $\dot{V}O_{2}$ , $\dot{V}CO_{2}$ , $\dot{V}E$ , and $V_{T}$ are recorded. Moreover, HR (typically ranging from 90–190 beats per minute) is recorded at each breath, yielding an additional 1500–2000 individual data points.

Despite the richness of these continuous breath-by-breath data, clinical practice generally refers to maximal, submaximal, or aggregated metrics (Leclerc, 2017; Kristenson et al., 2024). In particular, CPET in clinical practice has traditionally focused on $\dot{V}O_{2}$ peak. This emphasis originated from early exercise physiology work at the Harvard Fatigue Laboratory (Tipton, 1998; Bassett, 2002), where the primary goal was to quantify the maximal capacity of the cardiorespiratory system. Over time, $\dot{V}O_{2}$ peak remained the dominant measure, reinforced by its ease of computation, straightforward interpretation, and the extensive body of normative data supporting its use. Consequently, more complex analyses that could exploit the full time-series signal are rarely implemented in routine practice.

Some research has begun exploring methodologies to take advantage of the entire breath-by-breath data set from CPET (Cooper et al., 2014; Ntalianis et al., 2024; Ross et al., 2024). One promising approach involves the value of information science analytics such as entropy, a statistical measure that can quantify the complexity or unpredictability within time-series data. Entropy provides insights distinct from variability; for example, a highly variable but predictable signal (e.g., a sine wave) demonstrates low entropy. Entropy analysis may be especially informative in pediatric CPET as younger participants' breathing patterns tend to be more irregular than those of adults (Nagano et al., 1998; Ondrak and McMurray, 2006). This increased irregularity in breath timing and volume can produce greater fluctuations in the underlying time-series signal, enhancing the usefulness of entropy to detect differences in physiological control and adaptation during exercise.

Previous research (Blanks et al., 2024a; Blanks et al., 2024b) applied entropy analysis to pediatric populations, finding significant insights related to pubertal status and sex differences in CPET metrics. However, analyzing entropy of CPET variables presents unique challenges due to the short duration and non-stationary nature of the ramp CPET protocols where work rate and physiological signal responses change continuously without reaching a steady state. Non-stationarity violates assumptions of constant mean, variance, and autocorrelation, complicating entropy analysis. The method introduced in Blanks and Brown (2024) effectively addresses this by incorporating techniques to handle these non-stationary signals.

Building on this work, and continuing the process of validation of these new CPET analytics in the context of the growing child, the present study applies the entropy-based methodology used by Blanks et al. (2024b) to a larger pediatric data set (170 participants in this study vs. 81 in the previous study) with some key differences. For instance, the previous study separated the data at the ventilatory threshold $\left(VT_{1}\right)$ since progressive exercise testing results in at least two domains of physiological responses usually thought of as below or above the point during exercise at which lactate concentrations begin to increase in the circulating blood. However, the mechanism of this increase remains incompletely understood and consensus has yet to be established regarding what noninvasively obtained gas exchange and/or HR biomarker most accurately demarcates these exercise domains (Rossiter, 2021). The physiological regulation of heavier exercise is likely distinct as it is accompanied by the release of stress, inflammatory, immune, and other circulating mediators typically not increased in the central circulation at lighter exercise (Cooper et al., 2007). Precise measurements of any of the commonly accepted defining biomarkers is challenging in pediatric CPET given the increased breath-to-breath variability in gas exchange in younger compared to older children and young adults. Accordingly, we simplified our previous approach and performed our comparison of gas exchange before and after the midpoint of exercise duration. Additionally, we used age-cutoffs for comparisons as physical examination to determine pubertal status in younger participants and older participants is personally quite invasive and self-report is only moderately accurate (Rasmussen et al., 2015). Although $VT_{1}$ and Tanner stage provide physiologically meaningful cut points, both require subjective judgment and can be difficult to determine consistently. In contrast, age and test midpoint are simpler, more objective criteria that do not require additional clinical assessment. Robust data cleaning procedures were also implemented to minimize the impact of clear measurement errors on the results.

## 2 Materials and methods

### 2.1 Data description

#### 2.1.1 CPET

Children and adolescents were recruited for this study, which was approved by the University of California, Irvine Institutional Review Board. All procedures adhered to relevant ethical guidelines and regulations. Demographic and anthropometric measurements were collected using a calibrated scale and stadiometers. Participants then performed a CPET following an established ramp progressive protocol in the laboratory by pedaling on an electronically braked, servo-controlled cycle ergometer (Lode, Groningen, Netherlands) (Cooper et al., 2014). During testing, the Sensor Medics System (Vmax Encore 229, Yorba Linda, CA) collected gas exchange data and at each breath instantaneous HR was recorded. For consistency purposes, laboratory staff and faculty instructed and ensured participants maintained a pedaling rate between 65 and 75 revolutions per minute. Exercise continued until the participant or the supervising staff decided that the participant reached their limit of tolerance.

#### 2.1.2 Pediatric participant demographics and sample characteristics

This study included 170 participants aged 8–18 years (85 males) with no known cardiac, metabolic, or respiratory conditions and no history of taking medication for chronic disorders. The body mass index (BMI) of all participants fell between the 5.34% and 84.16% percentiles. [These BMI percentile values are sex-age specific, calculated utilizing CDC growth charts (Kuczmarski et al., 2002)]. The time series of breaths recorded during CPET for the 170 participants had a mean of 304.59±68.26 breaths per participant. All participants had a total test duration between 8 and 12 min and achieved a Respiratory Exchange Rate > 1.0. Breaths that met the criteria in Equation 1 were removed.

$$WR_{i,t}<\frac{WR_{i,95th}-WR_{i,5th}}{c_{i,95th}-c_{i,5th}}\left(c_{i,t}-1\right)$$ (1)

where i denotes participant i , t denotes observation or breath t , WR is the work rate in Watts, c is the time in minutes, $WR_{i,95th}$ , $c_{i,95th}$ , $WR_{i,5th}$ , and $c_{i,5th}$ represent the $95^{th}$ and $5^{th}$ percentiles of participant i ’s WR and c accordingly. To minimize the impact of erroneous data collection, we also removed extreme points, such as $\dot{V}O_{2}$ and $\dot{V}CO_{2}$ observations <0.2 L/min, HR observations that fell outside of the range 50−230 beats per minute, RR observations <8 and >75 breaths per minute, and $V_{T}$ observations <0.1 and >3.5 (L/breath). Additionally, we detected extreme points relative to surrounding points using a moving window and standard deviation process, as Hesse et al. (2025) found to be a common approach in CPET studies. Specifically, for each CPET metric m , let $b_{i,t}^{m}$ be the value for participant i at breath t . We defined the moving average for participant i at breath t in Equation 2.

$$\mu_{i,t}^{m}=\frac{1}{15}\overset{t+7}{\underset{j=t-7}{\sum }}b_{i,j}^{m}$$ (2)

Similarly, we defined the moving standard deviation at observation t in Equation 3.

$$\sigma_{i,t}^{m}=\sqrt{\frac{1}{15}\overset{t+7}{\underset{j=t-7}{\sum }}{\left(b_{i,j}^{m}-\mu_{i,t}^{m}\right)}^{2}}$$ (3)

Finally, we considered $b_{i,t}^{m}$ an extreme point (and subsequently removed from analysis) if it met the criteria of Equation 4.

$$\left|b_{i,t}^{m}-\mu_{i,t}^{m}\right|>3\sigma_{i,t}^{m}$$ (4)

After the mentioned data cleaning techniques, participants with missing breath-by-breath data for 30 consecutive seconds or more for any metric were excluded from this study. More participant information can be found in Table 1, where $\dot{V}O_{2}$ peak was calculated using the maximum 20 s rolling average, computed every 5 s of the last 2 min of exercise.

**TABLE 1:** Descriptive statistics of data set.

Metric	Males < 13	Males ≥ 13	Females < 12	Females ≥ 12
Count of participants	40	45	40	45
Age (years)	10.56±1.42	15.57±1.26	10.14±1.23	15.30±1.50
CPET duration (minutes)	9.71 ± 1.09	10.04 ± 0.95	9.29 ± 0.89	9.73 ± 0.91
BMI percentile	43.19 ± 23.67	47.06 ± 21.71	49.03 ± 23.98	43.76 ± 22.85
Height (cm)	142.08 ± 10.13	170.38 ± 7.60	142.43 ± 9.88	162.58 ± 7.13
Weight (kg)	34.44 ± 7.15	58.84 ± 8.18	35.04 ± 7.40	52.24 ± 6.41
$\dot{V}O_{2}$ peak (L/min)	1.47±0.29	2.59±0.55	1.27±0.27	1.90±0.44
$\dot{V}O_{2}$ peak (mL/min/kg)	43.42 ± 8.59	44.31 ± 8.34	36.71 ± 6.66	36.31 ± 7.84

To examine the effects of age and sex on Sample Entropy (SampEn), participants were first divided by sex into male and female groups. Within each sex, males were classified as younger than 13 years or older than 13 years, and females as younger than 12 years or older than 12 years. These cutoffs approximate the typical ages at which each sex reaches Tanner Stage 3 of 5 (Brix et al., 2019). Since this is an imperfect estimation, we also conducted additional analyses using an alternative female group cutoff at age 11 instead of 12 and excluding the 8 male participants between 12.5 (inclusive) and 13.5 years old (exclusive) and the 12 female participants between 11 (inclusive) and 12 (exclusive). The former provides some sensitivity analysis for the age delineator for female participants, and the latter creates more distinct age groups for both males and females. Minimal differences in results were observed between the groups. The results for these additional grouping mechanisms can be found in the Supplementary Data section. Since we created groups based off age, all results are interpreted as related to the age of the participants, and not to their maturational status.

### 2.2 Sample entropy and stationarity

The participants included in this study had a mean of 304.59±68.26 breaths (or distinct observations), which is typical in a progressive CPET. Each breath was considered a unique signal, and the timestamp was defined as the number of seconds elapsed since the CPET began. This posed a challenge, since information entropy analysis was originally designed for larger data sets. Information entropy analysis also assumes stationarity, which is not applicable to physiological metrics in a progressive CPET, since the mean of each metric increases as the test progresses. To solve these challenges, we performed the following SampEn related calculations, transformations, verifications, and decisions, by using “EristroPy” https://zblanks.github.io/eristropy. This Python programming package was designed specifically for the entropy analysis of short physiological signals and modifies the data to ensure weakly stationary signals (Blanks and Brown, 2024).

SampEn measures the uncertainty and predictability of dynamic time series data (Richman and Moorman, 2000). Let $x\in R^{N}$ define a time series signal of length N . The embedding dimension is an m -dimensional vector such that $x_{m}^{\left(i\right)}=\left(x_{i},x_{i+1},\ldots ,x_{i+m-1}\right)$ . The distance between templates is evaluated in relation to a predefined similarity radius, r>0 , as outlined in Equation 5.

$$∥x_{m}^{\left(i\right)}-x_{m}^{\left(j\right)}{∥}_{\infty }=\underset{k=1,\ldots ,m}{\max}|x_{m}^{\left(i\right)}\left(k\right)-x_{m}^{\left(j\right)}\left(k\right)|\leq r,$$ (5)

where $x_{m}^{\left(i\right)}\left(k\right)$ is the k -th element of the vector. This process repeats for all combinations except for self-checks. $B^{m}\left(r\right)$ denotes the probability that the time series data remains within a distance r for m points. See Equation 6.

$$B^{m}\left(r\right)=\frac{1}{Z\left(N,m\right)}\overset{N-m}{\underset{i=1}{\sum }}\overset{N-m-1}{\underset{j=1,i\neq j}{\sum }}\Theta \left[∥x_{m}^{\left(i\right)}-x_{m}^{\left(j\right)}{∥}_{\infty }\leq r\right],$$ (6)

where Θ[⋅] represents the Heaviside function, evaluating to 1 when the condition is true and 0 otherwise. Moreover, Z(N,m) is the normalization constant so that $B^{m}\left(r\right)$ always lies between 0 and 1.

To compute the probability that the time series data remain within a distance r for m+1 points, see Equation 7 below.

$$A^{m}\left(r\right)=\frac{1}{Z\left(N,m\right)}\overset{N-m}{\underset{i=1}{\sum }}\overset{N-m-1}{\underset{j=1,i\neq j}{\sum }}\Theta \left[∥x_{m+1}^{\left(i\right)}-x_{m+1}^{\left(j\right)}{∥}_{\infty }\leq r\right].$$ (7)

Finally, SampEn can be calculated using Equation 8.

$$SampEn\left(x,m,r\right)=-log\frac{A^{m}\left(r\right)}{B^{m}\left(r\right)}.$$ (8)

Richman and Moorman (2000) provides details of the mathematical foundations of SampEn. Chatain et al. (2020) discusses that it is vital to work with stationary signals for accurate SampEn analysis. To account for CPET signals being non-stationary, we modified the signals into a weakly stationary form by applying a differencing algorithm. In particular, if x is a CPET signal, the differenced signal can be represented as $\tilde{x}$ defined in Equation 9.

$$\tilde{x_{t}}=x_{t}-x_{t-1},\;t=2,\ldots ,N,$$ (9)

where N represents the total number of observations in x . Then, the updated set of signals were standardized to have zero mean and unit variance. Next, these standardized and updated signals were checked for weakly stationary signals at a significance level of α=0.05 , leveraging the Augmented Dickey and Fuller test. After the significance level assessment, multiple testing errors were corrected using the Holm-Sidak method. Optimal SampEn parameters ( m , r ) were selected using the method explained in Blanks and Brown (2024). It is worth noting that the optimal m and r were found independently for each metric using all participants, regardless of age or sex. The penalization parameter on r , lam , was set to 0.2 for metrics $\dot{V}O_{2}$ , $\dot{V}CO_{2}$ , $\dot{V}E$ , RR , and $V_{T}$ , and to 0.006 for HR (to guarantee proper exploration of the parameter search space). See Blanks and Brown (2024) for information on lam selection and “EristroPy” documentation for more details on the overall approach to SampEn https://zblanks.github.io/eristropy.

### 2.3 SampEn Pre- and post- midpoint

Blanks et al. (2024b) also studied the SampEn of each participant before and after they reached their $VT_{1}$ . Blanks et al. (2024b) estimated $VT_{1}$ using the process used by Kim et al. (2021), where they defined $VT_{1}$ to be the average of the $VT_{1}$ detected via the V-slope and excess $CO_{2}$ methods. When applying this process to the present study’s data set, we found notable differences in $VT_{1}$ detected using the V-slope approach compared to the excess $CO_{2}$ approach, introducing uncertainty around the participants’ true $VT_{1}$ . To avoid analysis based on incorrect $VT_{1}$ detection, we compared entropy before and after the midpoint of the test. We defined the midpoint for each participant as their total CPET time divided in half. Any breath observed before the halfway mark of the total time of the CPET was labeled as “pre-midpoint,” and any breath observed at or after the halfway mark was labeled as “post-midpoint.”

### 2.4 Bayesian statistical testing

To analyze differences in the entropy of the gas exchange and HR variables across sexes, age groups, and midpoint status, we implemented Bayesian models defined with the following notation:

-
M denotes the set of CPET metrics { $\dot{V}O_{2}$ , $\dot{V}CO_{2}$ , $\dot{V}E$ , HR , RR , $V_{T}$ }.
-
S denotes the set {Male, Female}
-
A denotes the set of age groups {Child, Adolescent}
-
H denotes the set of midpoint status {Pre-Midpoint, Post-Midpoint}.
-
I(m,s,a,h) denotes the set of indices corresponding to participants with a CPET metric m , sex s , age group a , with midpoint status h .
-
I(m,s,a) denotes the set of indices corresponding to participants with a CPET metric m , sex s , age group a .

The following specifications were placed on the model:

-
$\mu_{s,a,h}^{\left(m\right)}∼N\left(2,1\right)$

-
$\sigma_{s,a,h}^{\left(m\right)}∼U\left(0.05,0.50\right)$

• ν∼logN(1,1)

-
$y_{i,s,a,h}^{\left(m\right)}∼t\left(\mu_{s,a,h}^{\left(m\right)},\sigma_{s,a,h}^{\left(m\right)},\nu \right)$

-
$\mu_{s,a}^{\left(m\right)}∼N\left(2,1\right)$

-
$\sigma_{s,a}^{\left(m\right)}∼U\left(0.05,0.50\right)$

-
$y_{i,s,a}^{\left(m\right)}∼t\left(\mu_{s,a}^{\left(m\right)},\sigma_{s,a}^{\left(m\right)},\nu \right)$

These prior distributions and parameter decisions are the same as those in Blanks et al. (2024b) for effective comparison of SampEn results. Specifically, the Gaussian prior for μ and the uniform prior for σ represent the reasonably bounded range of entropy observed in healthy younger participants within this context. The prior on μ has a large variance to account for the differences between gas exchange entropy and HR entropy. The heavy-tailed distribution of ν accommodates potential extreme points in the data set.

We utilized PyMC Abril-Pla et al. (2023) for Markov chain Monte Carlo (MCMC) sampling via the No U-Turn Sampler algorithm. For each sample, 4000 posterior draws were made from MCMC. We evaluated convergence using trace plots, effective sample size, and the $\hat{R}$ statistic (Vehtari et al., 2021).

#### 2.4.1 Midpoint entropy differences

To compare SampEn pre- and post-midpoint, we defined $\mu_{s,a,h}^{m}$ as the inferred mean SampEn for participants of sex s (0 = male, 1 = female), age group a (0 = younger, 1 = older), midpoint status h (0 = pre-midpoint, 1 = post-midpoint) for metric m . The change in SampEn from pre-to post-midpoint was then computed using Equation 10.

$$\Delta \mu_{s,a}^{\left(m,mid\right)}=\mu_{s,a,1}^{m}-\mu_{s,a,0}^{m}.$$ (10)

The estimated probability that $\Delta \mu_{s,a}^{\left(m,mid\right)}$ is greater than or equal to zero is represented in Equation 11.

$$P\left(\Delta \mu_{s,a}^{\left(m,mid\right)}\geq 0|y\right)\approx \frac{1}{D}\overset{D}{\underset{k=1}{\sum }}1\left[\Delta \mu_{s,a}^{\left(m,mid\right)}\left(k\right)\geq 0\right],$$ (11)

based on D=4000 posterior draws from MCMC, where 1[⋅] denotes the indicator function. Thus, when $P\left(\Delta \mu_{s,a}^{\left(m,mid\right)}\geq 0|y\right)<0.5$ , there are more instances of lower SampEn post-midpoint than pre-midpoint of CPET. We set the threshold for all analyses such that if $P\left(\Delta \mu_{s,a}^{\left(m,mid\right)}\geq 0|y\right)<0.05$ or $P\left(\Delta \mu_{s,a}^{\left(m,mid\right)}\geq 0|y\right)>0.95$ then there is statistical significance that SampEn is higher pre-midpoint or higher post-midpoint, respectively.

#### 2.4.2 Total entropy analysis: effect of age on SampEn

In this section, we examined the entropy differences across the age groups for the entire CPET, regardless of the midpoint. We evaluated the following mean posterior SampEn percentage difference between the older participants and the younger participants in Equation 12.

$$\Delta \mu_{s}^{\left(m\right)}=\frac{\mu_{s,1}^{\left(m\right)}-\mu_{s,0}^{\left(m\right)}}{\mu_{s,1}^{\left(m\right)}}\times 100,$$ (12)

where $\mu_{s,a}^{\left(m\right)}$ represents the mean posterior SampEn estimate for CPET metric m for sex s (0 = male, 1 = female) and age group a (0 = younger, 1 = older). Therefore, a negative $\Delta \mu_{s}^{\left(m\right)}$ supports the claim that younger participants on average have higher SampEn for CPET metrics than older participants.

The estimated probability that $\Delta \mu_{s}^{\left(m\right)}$ is greater than zero is represented in Equation 13:

$$P\left(\Delta \mu_{s}^{\left(m\right)}\geq 0|y\right)\approx \frac{1}{D}\overset{D}{\underset{k=1}{\sum }}1\left[\Delta \mu_{s}^{\left(m\right)}\left(k\right)\geq 0\right],$$ (13)

based on D=4000 posterior draws from MCMC, where 1[⋅] denotes the indicator function. Therefore, $P\left(\Delta \mu_{s}^{\left(m\right)}\geq 0|y\right)<0.5$ indicates that there are more instances of higher SampEn for the younger participants. Similar to the midpoint analysis, for our purposes, $P\left(\Delta \mu_{s}^{\left(m\right)}\geq 0|y\right)<0.05$ or $P\left(\Delta \mu_{s}^{\left(m\right)}\geq 0|y\right)>0.95$ indicate that statistical significance that SampEn is higher for younger participants or higher for older participants, respectively.

#### 2.4.3 Total entropy analysis: effect of sex on SampEn

As a part of this study, we also explored the entropy differences between males and females for the entire CPET, regardless of the midpoint. For each CPET metric m , we evaluated the mean posterior SampEn percentage difference using Equation 14.

$$\Delta \mu_{a}^{\left(m\right)}=\frac{\mu_{0,a}^{\left(m\right)}-\mu_{1,a}^{\left(m\right)}}{\mu_{1,a}^{\left(m\right)}}\times 100$$ (14)

where $\mu_{s,a}^{\left(m\right)}$ represents the mean posterior SampEn estimate for CPET metric m for participants of sex s (0 = male, 1 = female) and age group a (0 = younger, 1 = older). Therefore, a negative $\Delta \mu_{a}^{\left(m\right)}$ supports the findings in Blanks et al. (2024a) and Blanks et al. (2024b) that pediatric females on average have higher SampEn for CPET metrics than pediatric males.

The estimated probability that $\Delta \mu_{a}^{\left(m\right)}$ is greater than or equal to zero is represented in Equation 15.

$$P\left(\Delta \mu_{a}^{\left(m\right)}\geq 0|y\right)\approx \frac{1}{D}\overset{D}{\underset{k=1}{\sum }}1\left[\Delta \mu_{a}^{\left(m\right)}\left(k\right)\geq 0\right],$$ (15)

based on D=4000 posterior draws from MCMC, where 1[⋅] denotes the indicator function. If $P\left(\Delta \mu_{a}^{\left(m\right)}\geq 0|y\right)<0.5$ , then there are more instances of higher SampEn for female participants. Again, if $P\left(\Delta \mu_{a}^{\left(m\right)}\geq 0|y\right)<0.05$ or $P\left(\Delta \mu_{a}^{\left(m\right)}\geq 0|y\right)>0.95$ are the thresholds for statistical significance that SampEn is higher for female participants or higher for male participants, respectively.

## 3 Results

### 3.1 Midpoint entropy differences

**FIGURE 1:**
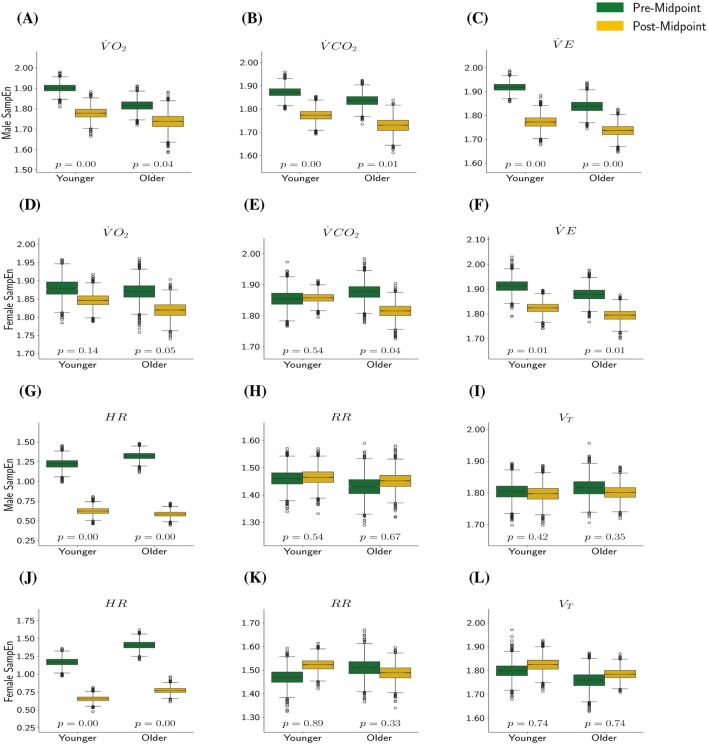
SampEn Pre- and Post-Midpoint by Metric, Sex, and Age. In this plot, p represents $P\left(\Delta \mu_{s,a}^{\left(m\right)}\geq 0|y\right)$ , with p<0.05 or p>0.95 indicating a statistical significance that SampEn (the measure of complexity) is higher pre-midpoint or higher post-midpoint, respectively.

Similar to the post $VT_{1}$ results reported in Blanks et al. (2024b), we hypothesized that SampEn would decrease after the midpoint of the CPET for the metrics $\dot{V}O_{2}$ , $\dot{V}CO_{2}$ , $\dot{V}E$ , and HR , and increase for RR and ${\dot{V}}_{T}$ across all combinations of sex and age groups. The results in Figure 1 indicate that:

- For all four combinations of age and sex, (except for younger females’ $\dot{V}CO_{2}$ ) there is an average decrease in SampEn after the midpoint of the CPET for the gas exchange metrics $\dot{V}O_{2}$ , $\dot{V}CO_{2}$ , $\dot{V}E$ , and HR , with HR having the most pronounced decrease. This decrease is strongly supported (with $P\left(\Delta \mu_{s,a}^{\left(m\right)}\geq 0|y\right)<0.05$ ) for younger male participants and older male participants, for all four metrics, for younger female participants for $\dot{V}E$ and HR , and for older females for $\dot{V}CO_{2}$ , $\dot{V}E$ , and HR

- The differences between RR and $V_{T}$ pre- and post-midpoint for all four age and sex groups lacks statistical significance.

### 3.2 Total entropy analysis

In this section, we examine the differences in entropy between sexes and age groups for the entire CPET, regardless of the midpoint.

#### 3.2.1 Effect of age on SampEn

**FIGURE 2:**
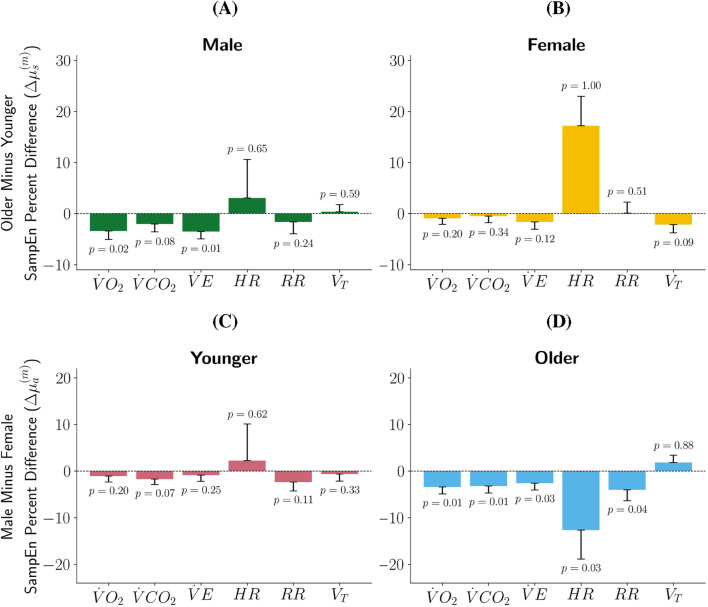
Percent Difference in SampEn by Sex and Age Group. In plots **(A,B)** p represents $P\left(\Delta \mu_{s}^{\left(m\right)}\geq 0|y\right)$ , with p<0.05 or p>0.95 indicating statistical significance that SampEn (the measure of complexity) is higher for younger participants or higher for older participants, respectively. In plots **(C,D)** p represents $P\left(\Delta \mu_{a}^{\left(m\right)}\geq 0|y\right)$ , with p<0.05 or p>0.95 indicating statistically significance, with SampEn (the measure of complexity) being higher for female participants or higher for male participants, respectively. The further p is from 0.5, the more statistically significant the mean percent difference between the two data sets. The length of the bars represents $\Delta \mu_{s}^{\left(m\right)}$ [plots **(A,B)**] and $\Delta \mu_{a}^{\left(m\right)}$ [plots **(C,D)**] and the black represents one standard deviation of the posterior SampEn estimates for CPET metric m for participants of sex s [plots **(A,B)**] and age group a [plots **(C,D)**].

Results and analysis detailed in Blanks et al. (2024a) and Blanks et al. (2024b) both suggest that early-pubertal participants tend to have higher SampEn than late-pubertal participants, particularly female participants. Thus, we hypothesized that the younger participants would have higher SampEn than the older participants in our sample. Recall that if $P\left(\Delta \mu_{s}^{\left(m\right)}\geq 0|y\right)<0.05$ or $P\left(\Delta \mu_{s}^{\left(m\right)}\geq 0|y\right)>0.95$ , then it is statistically significant that SampEn is higher for younger participants or higher for older participants, respectively, for the given metric m . Figures 2A,B presents the following:

- Younger male participants have higher SampEn than older male participants with statistical significance for $\dot{V}O_{2}$ and $\dot{V}E$ .
- Older male participants have higher SampEn than younger male participants for HR and $V_{T}$ but without statistical significance.
- Younger female participants have a higher SampEn than older female participants without statistical significance for metrics $\dot{V}O_{2}$ , $\dot{V}CO_{2}$ , $\dot{V}E$ , and $V_{T}$ .
- Older female participants have a higher SampEn than younger female participants for HR with statistical significance. The height of this bar is remarkably taller than the others; this indicates that the percent difference between younger female participants and older participants’ raw HR SampEn is large in addition to statistically significant.

#### 3.2.2 Effect of sex on SampEn

Blanks et al. (2024a) and Blanks et al. (2024b) found that pediatric female participants tend to exhibit greater SampEn of CPET metrics than pediatric male participants. We aimed to determine if this claims extends to the participants in this study. Results shown in Figures 2C,D indicate:

- For the younger participants, females have a higher SampEn for $\dot{V}O_{2}$ , $\dot{V}CO_{2}$ , $\dot{V}E$ , RR , and $V_{T}$ , but without statistical significance.
- Younger male participants have higher SampEn for HR than younger female participants without statistical significance.
- Older females have statistically significant higher SampEn than their male counterparts for $\dot{V}O_{2}$ , $\dot{V}CO_{2}$ , $\dot{V}E$ , HR , and RR . The percent difference for HR between adolescent males and females has higher magnitude.
- Older males have higher SampEn than their female counterparts for $V_{T}$ without statistical significance.

### 3.3 Comparison to previous study

**FIGURE 3:**
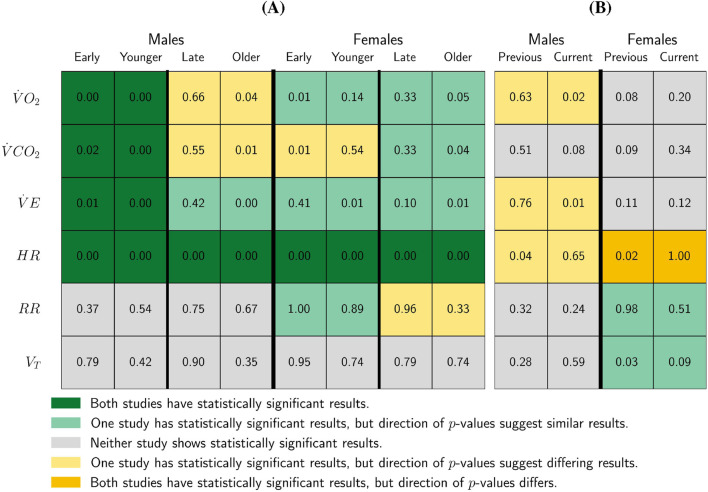
Study Comparison of SampEn Pre-/Post- $VT_{1}$ and Midpoint and Study Comparison of SampEn Early Puberty/Child and Late Puberty/Adolescent. Recall that the previous study analyzed SampEn (the measure of complexity) pre-/post- $VT_{1}$ , and in the current study, we analyzed SampEn pre-/post-midpoint. Additionally, the previous study segmented groups based on pubertal status (early or late) and in the current study, we segmented groups based on age (child or adolescent). The values in plot **(A)** represent $P\left(\Delta \mu_{s,a}^{\left(m\right)}\geq 0|y\right)$ with a value <0.05 or >0.95 indicating a statistical significance that SampEn (the measure of complexity) is higher pre- $VT_{1}$ /pre-midpoint or higher post- $VT_{1}$ /post-midpoint, respectively. Meanwhile, the values in plot **(B)** are $P\left(\Delta \mu_{s}^{\left(m\right)}\geq 0|y\right)$ , with <0.05 or >0.95 indicating statistical significance that SampEn (the measure of complexity) is higher for younger participants or higher for older participants, respectively.

We compared these results to those reported in Blanks et al. (2024b) (Figure 3). In that study, participants were grouped by pubertal status, while in this study, we used age. For direct comparison, we aligned Blanks et al. (2024b) early pubertal participants with the younger participants in this study, and the late pubertal participants with our older participants. Note that the same statistical procedures and significance thresholds were used in both studies.

Key points from Figure 3 include:

- Consistent findings across studies: Both studies find statistically significant decreases in SampEn post- $VT_{1}$ or post-midpoint for $\dot{V}O_{2}$ , $\dot{V}CO_{2}$ , and $\dot{V}E$ for the male early-pubertal and younger participants groups, and for HR in all four sex and pubertal status/age groups.

Differences in statistical significance: Although the direction of results was generally consistent, statistical significance varied. Only one study presented statistically significant results for:

- A decline in SampEn of $\dot{V}E$ in older male participants [late-pubertal females in Blanks et al. (2024b)].
- A decline in SampEn of $\dot{V}O_{2}$ and $\dot{V}E$ , and an increase in SampEn of RR for younger female participants [early-pubertal females in Blanks et al. (2024b)].
- A decline in SampEn for $\dot{V}O_{2}$ , $\dot{V}CO_{2}$ , and $\dot{V}E$ for older female participants [late-pubertal females in Blanks et al. (2024b)].

- Male adolescent differences: The present study finds a statistically significant decline in SampEn post-midpoint for older male participants, whereas Blanks et al. (2024b) observed a non-significant increase in $\dot{V}O_{2}$ and $\dot{V}CO_{2}$ in late pubertal males post-the $VT_{1}$ of the CPET.
- Younger female $\dot{V}CO_{2}$ differences: Blanks et al. (2024b) found a statistically significant decline in SampEn post- $VT_{1}$ for early-pubertal females, while this study finds a non-significant subtle increase post-midpoint.
- Older female RR differences: Blanks et al. (2024b) reported a statistically significant increase in SampEn post- $VT_{1}$ for late-pubertal females’ RR metric, whereas our results showed a non-significant decrease.

Figure 3 also compares the $P\left(\Delta \mu_{s}^{\left(m\right)}\geq 0|y\right)$ of both studies, which represents the probability that the differences between late and early pubertal participants in Blanks et al. (2024b) and older participants and younger participants in this study are greater than 0 by sex.

- Both the present study and Blanks et al. (2024b) younger female participants (early-puberty) to have higher SampEn than their older (late-puberty) counterparts for the $V_{T}$ metric. However, only Blanks et al. (2024b) found each of this difference to be statistically significant.
- Both studies found older female participants (late-puberty) to have higher SampEn than younger female participants (early-puberty) for the RR metric, but only Blanks et al. (2024b) found this differences to be statistically significant.
- For males, the current study found the younger participants to have higher SampEn than the older participants for $\dot{V}O_{2}$ and $\dot{V}E$ with statistical significance, but Blanks et al. (2024b) found insignificant results in the opposite direction between early- and late-pubertal participants.
- Blanks et al. (2024b) found younger male participants (early-puberty) to have statistically significantly higher SampEn than their older male (late-puberty) counterparts for the HR metric, but the current study found insignificant results in the other direction.
- The current work found older female participants to have higher HR SampEn than younger female participants with statistically significance. However, Blanks et al. (2024b) found statistical significance in early-pubertal females having higher HR SampEn than late-pubertal females.

## 4 Discussion

This study strengthens the findings of Blanks et al. (2024b) regarding how SampEn changes as exercise intensity increases during CPET. Specifically, SampEn consistently decreases for $\dot{V}O_{2}$ , $\dot{V}CO_{2}$ , $\dot{V}E$ , and HR as exercise intensity increases in the latter half of the CPET. These statistically significant results validate entropy of breath-by-breath time series data as a way to measure and understand people’s physiological response to exercise.

The consistent decreases in SampEn for $\dot{V}O_{2}$ , $\dot{V}CO_{2}$ , $\dot{V}E$ , and HR after the CPET midpoint aligns with the findings of Blanks et al. (2024b), suggesting that $\dot{V}O_{2}$ , $\dot{V}CO_{2}$ , $\dot{V}E$ , and HR become more predictable as exercise intensity increases, independent of age or sex. This general agreement across studies provides additional evidence of increased predictability in key gas exchange variables during high intensity exercise in pediatric participants. Similar patterns have been reported in other physical domains; for example, Solís-Montufar et al. (2020) reported a decline in the entropy of the electrocardiogram RR interval as exercise intensity increased for the young and middle-aged adults in their study. Collectively, the consistent decrease in entropy observed after the CPET midpoint may reflect the physiological changes that occur as participants progress toward maximal exercise intensity. Per Pincus (1994), reductions in approximate entropy indicate higher predictability, interpreted as greater component isolation. Jiang et al. (2021) reported an increase in entropy of $SpO_{2}$ during normobaric hypoxia, while the current work shows decreased entropy as metabolic and ventilatory demands reach maximum levels. These findings support SampEn as a non-invasive marker of cardiorespiratory dynamics under stress, which can be applied to monitor physical health and aerobic fitness during exercise testing.

In this study, females had higher SampEn than males in both age groups for $\dot{V}O_{2}$ , $\dot{V}CO_{2}$ , $\dot{V}E$ , and RR . Previous studies (Blanks et al., 2024a; Blanks et al., 2024b) also found higher SampEn in females for CPET metrics, although those differences were not always statistically significant. The current work found statistically significant differences between the SampEn of adolescent males and females for $\dot{V}O_{2}$ , $\dot{V}CO_{2}$ , $\dot{V}E$ , HR , and RR , adding evidence to the suggestion of possible sex-related differences in ventilatory control or metabolic regulation discussed in Dominelli and Molgat-Seon (2022) that warrant further physiological investigation.

One limitation of this work relates specifically to the HR data. Note that in Figure 1 the SampEn of HR is lower than the other gas exchange variables, particularly post-midpoint. In CPET, HR is measured continuously via ECG or heart rate monitor but stored in the breath-by-breath dataset by assigning an HR value to each recorded breath. Because there are typically more breaths in the latter half of the test, this approach results in more repeated HR values in that segment, which may increase redundancy in the HR signal and incidentally lower SampEn post-midpoint. Nonetheless, the substantial pre–post midpoint differences in HR entropy remained statistically significant. We surmise that the lower entropy of HR observed during heavier exercise, compared with gas exchange variables, emerges because HR is virtually controlled by autonomic mechanisms (Fukumoto et al., 2022), while breathing is under the control of both involuntary and voluntary regulatory mediators (Grassmann et al., 2016).

As a part of this experiment, the SampEn of the differenced signal was calculated to produce a weakly stationary form. This step is necessary as Chatain et al. (2020) found that failing to account for the non-stationarity of physiological force signals increases SampEn. Differencing the signal emphasizes short-term fluctuations versus the gradually increasing trend of CPET metrics over time.

We also chose to group participants by age rather than pubertal status. This decision was intentional, as age is an objective and easily collected measure, whereas pubertal status is more difficult to obtain (Ernst et al., 2018; Ebo et al., 2024). While age may not perfectly align with maturational status, our sensitivity analyses using alternative age cutoffs and excluding participants near the thresholds produced minimal differences in results. This supports the suitability of age as a practical proxy in this context, while acknowledging that maturational status can provide additional developmental insight.

Finally, we segmented the CPET at the midpoint rather than $VT_{1}$ . In pediatric data analysis, the midpoint offers a simpler and more consistent approach, avoiding the methodological variability and interpretation challenges associated with $VT_{1}$ determination in younger participants. Although this choice sacrifices some individualized metabolic information, it improves standardization across participants and facilitates reproducible analysis.

The stark disagreement between this study and (Blanks et al., 2024b) for the female age and pubertal status groups’ HR metric also warrants further investigation. Possible explanations include differences in data processing and modifications made to satisfy SampEn assumptions. While we are confident that all data processing implemented in this work was necessary, we are uncertain how or if these steps affected the SampEn results. Future research on best practices for CPET data processing is essential, particularly given that Hesse et al. (2025) found only 376 out of 8,344 of articles reviewed mentioned removing outliers. The contrasts of these studies may also suggest that maximal CPET (with short signals and lack of stationarity without modifications) may not provide optimal data to measure SampEn of gas exchange metrics and HR for comparison between maturational statuses, ages, or sex during exercise. This area also requires further research.

In conclusion, this study builds on the work of Blanks et al. (2024b) by applying entropy-based analysis to a larger pediatric CPET cohort. We confirmed consistent post-midpoint declines in $\dot{V}O_{2}$ , $\dot{V}CO_{2}$ , $\dot{V}E$ , and HR , supporting SampEn as a promising tool for capturing physiological changes during high-intensity exercise. We also identified significant sex differences in SampEn for multiple CPET variables. Our methodological choices, using age as a practical proxy for maturational status, segmenting tests at the midpoint for consistency, and implementing robust data processing procedures, were intentional and supported by sensitivity analyses showing minimal impact on results. While certain discrepancies with previous work highlight the need for standardized CPET data processing practices and further evaluation of SampEn in pediatric exercise testing, the present findings strengthen the evidence for its utility as a non-invasive measure of physiological response across age and sex groups.

## Data Availability

The data analyzed in this study is subject to the following licenses/restrictions: The datasets presented in this article are not readily available because human subjects’ data is protected beyond the research team. Requests to access these datasets should be directed to Shlomit Radom Aizik, saizik@hs.uci.edu.

## Supplementary material

SUPPLEMENTARY FIGURE S1 SampEn Pre- and Post-Midpoint by Metric, Sex, and Age - Female Age Cut Off of 11 Years. In this plot, p represents $P\left(\Delta \mu_{s,a}^{\left(m\right)}\geq 0|y\right)$ , with p<0.05 or p>0.95 indicating a statistical significance that SampEn (the measure of complexity) is higher pre-midpoint or higher post-midpoint, respectively.

SUPPLEMENTARY FIGURE S2 SampEn Pre- and Post-Midpoint by Metric, Sex, and Age - Remove Middle Aged Male and Female Participants. In this plot, p represents $P\left(\Delta \mu_{s,a}^{\left(m\right)}\geq 0|y\right)$ , with p<0.05 or p>0.95 indicating a statistical significance that SampEn (the measure of complexity) is higher pre-midpoint or higher post-midpoint, respectively.

SUPPLEMENTARY FIGURE S3 Percent Difference in SampEn by Sex and Age Group - Female Age Cut Off of 11 Years. In plots **(A,B)** p represents $P\left(\Delta \mu_{s}^{\left(m\right)}\geq 0|y\right)$ , with p<0.05 or p>0.95 indicating statistical significance that SampEn (the measure of complexity) is higher for younger participants or higher for older participants, respectively. In plots **(C,D)** p represents $P\left(\Delta \mu_{a}^{\left(m\right)}\geq 0|y\right)$ , with p<0.05 or p>0.95 indicating statistically significance, with SampEn (the measure of complexity) being higher for female participants or higher for male participants, respectively. The further p is from 0.5, the more statistically significant the mean percent difference between the two data sets. The length of the bars represents $\Delta \mu_{s}^{\left(m\right)}$ [plots **(A,B)**] and $\Delta \mu_{a}^{\left(m\right)}$ [plots **(C,D)**] and the black represents one standard deviation of the posterior SampEn estimates for CPET metric m for participants of sex s [plots **(A,B)**] and age group a [plots **(C,D)**].

SUPPLEMENTARY FIGURE S4 Percent Difference in SampEn by Sex and Age Group - Remove Middle Aged Male and Female Participants. In plots **(A,B)** p represents $P\left(\Delta \mu_{s}^{\left(m\right)}\geq 0|y\right)$ , with p<0.05 or p>0.95 indicating statistical significance that SampEn (the measure of complexity) is higher for younger participants or higher for older participants, respectively. In plots **(C,D)** p represents $P\left(\Delta \mu_{a}^{\left(m\right)}\geq 0|y\right)$ , with p<0.05 or p>0.95 indicating statistically significance, with SampEn (the measure of complexity) being higher for female participants or higher for male participants, respectively. The further p is from 0.5, the more statistically significant the mean percent difference between the two data sets. The length of the bars represents $\Delta \mu_{s}^{\left(m\right)}$ [plots **(A,B)**] and $\Delta \mu_{a}^{\left(m\right)}$ [plots **(C,D)**] and the black represents one standard deviation of the posterior SampEn estimates for CPET metric m for participants of sex s [plots **(A,B)**] and age group a [plots **(C,D)**].
