# Supplementary figures and images for: Entropy as a marker of physiological transition during pediatric cardiopulmonary exercise testing
Source: Front Physiol. 2025 Dec 1;16:1698399. doi: 10.3389/fphys.2025.1698399 (PMC12702724; doi:10.3389/fphys.2025.1698399)

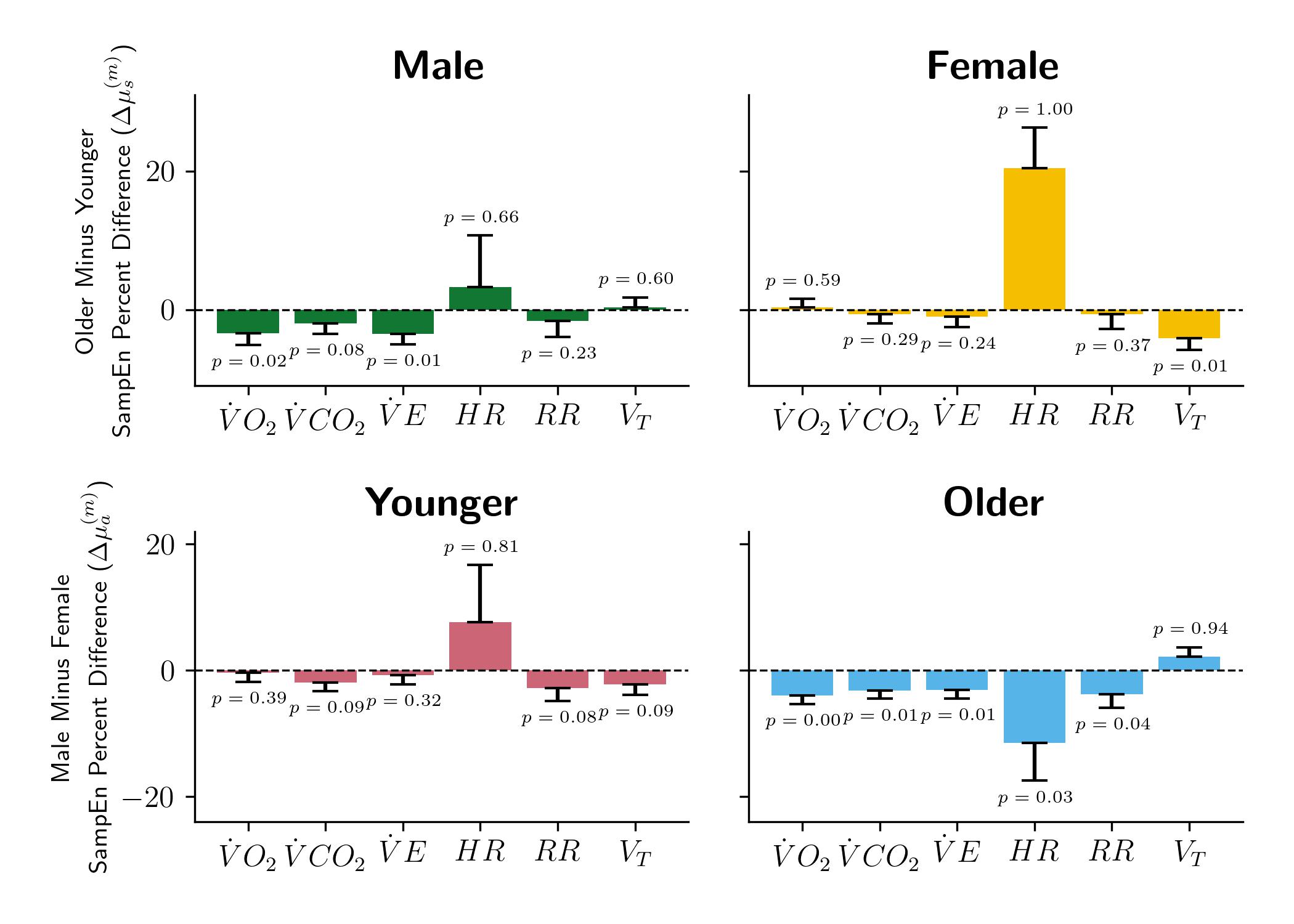

Supplement: Supplementary file 1 [file Image3.jpeg]

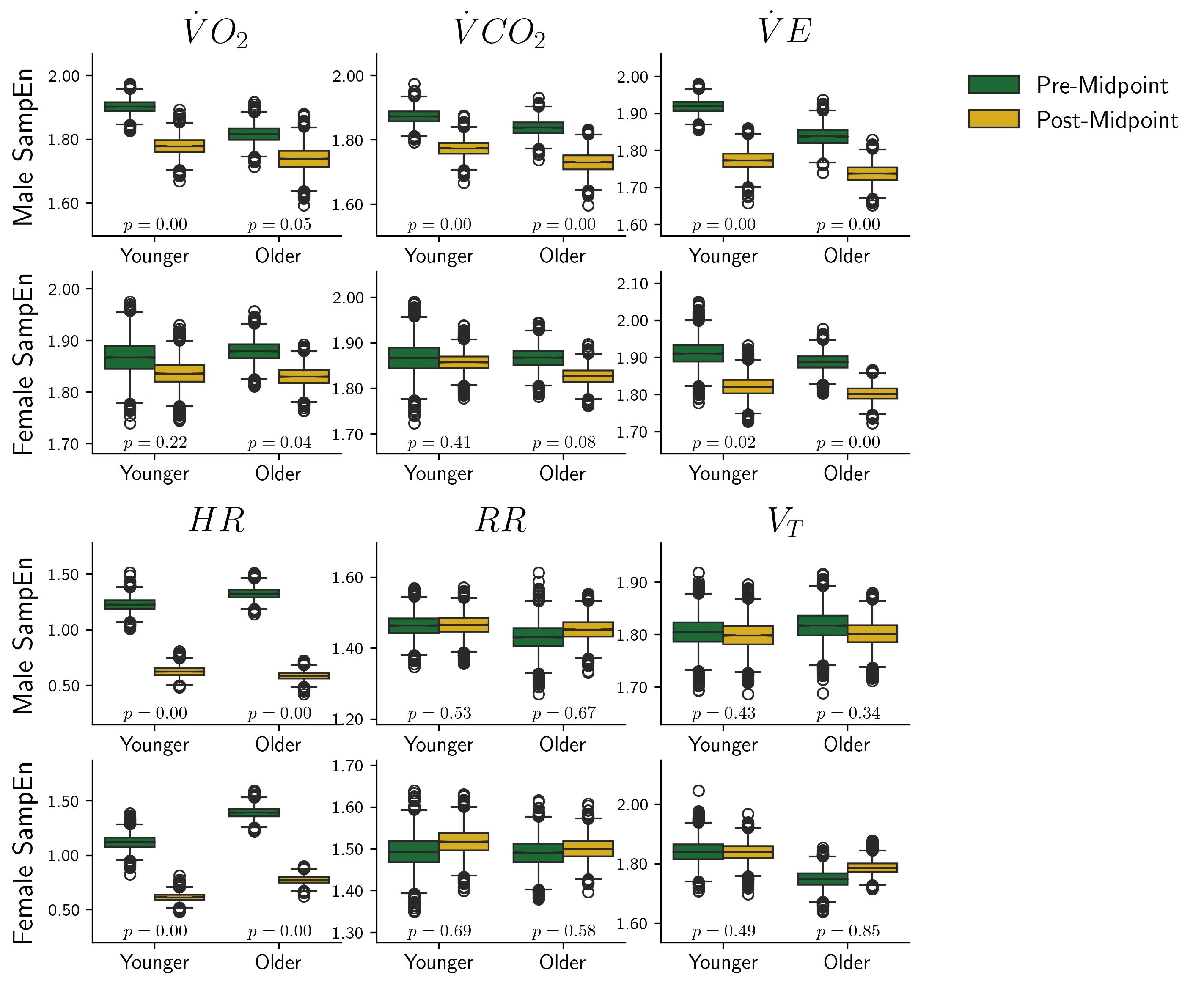

Supplement: Supplementary file 2 [file Image1.jpeg]

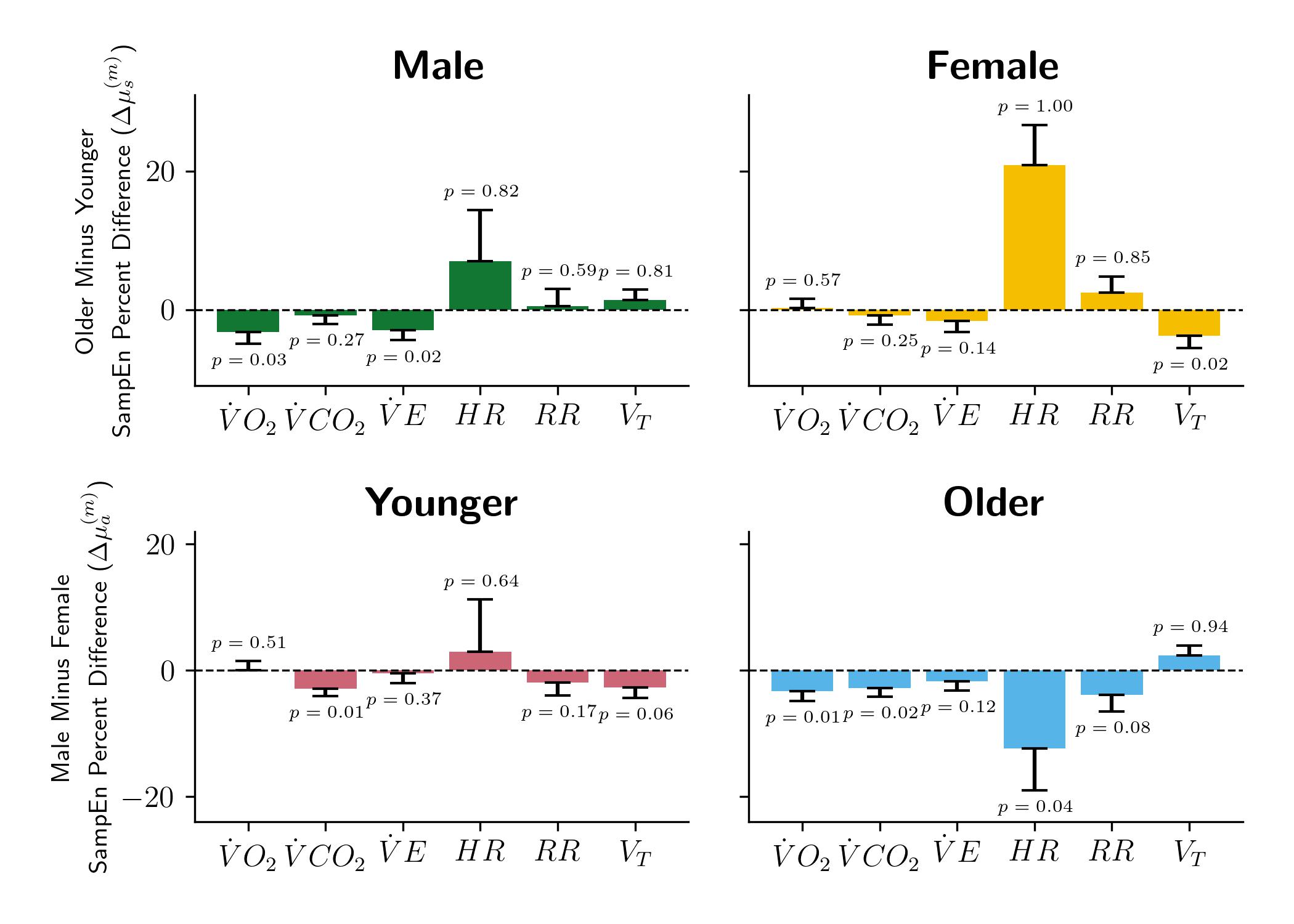

Supplement: Supplementary file 3 [file Image4.jpeg]

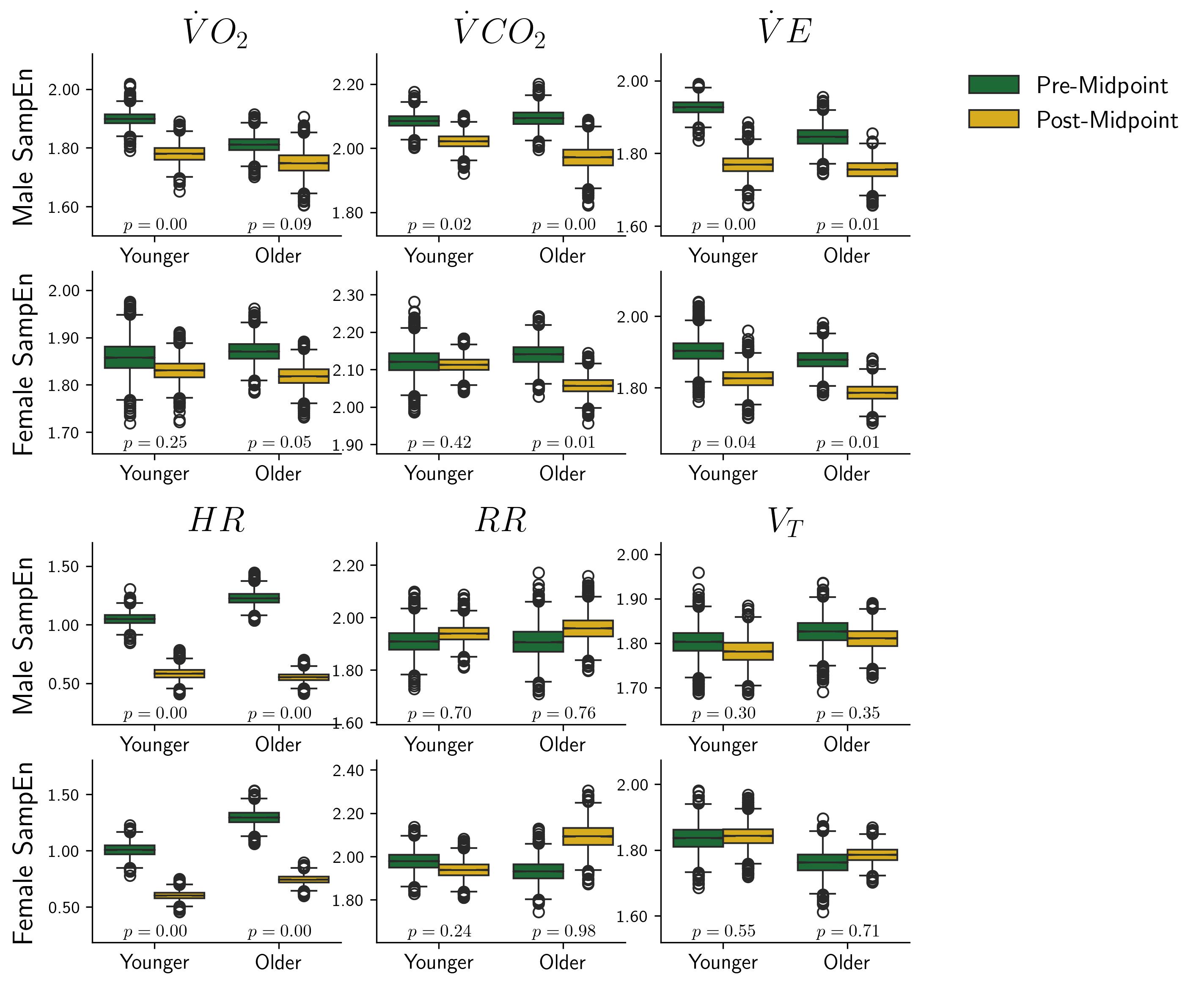

Supplement: Supplementary file 4 [file Image2.jpeg]
